# The Effect of Sage (*Salvia sclarea*) Essential Oil on the Physiochemical and Antioxidant Properties of Sodium Alginate and Casein-Based Composite Edible Films

**DOI:** 10.3390/gels9030233

**Published:** 2023-03-16

**Authors:** Saurabh Bhatia, Ahmed Al-Harrasi, Yasir Abbas Shah, Muhammad Jawad, Mohammed Said Al-Azri, Sana Ullah, Md Khalid Anwer, Mohammed F. Aldawsari, Esra Koca, Levent Yurdaer Aydemir

**Affiliations:** 1 Natural and Medical Sciences Research Center, University of Nizwa, P.O. Box 33, Birkat Al Mauz, Nizwa 616, Oman; yasir.shah@unizwa.edu.om (Y.A.S.); m.jawad@unizwa.edu.om (M.J.); malazri@unizwa.edu.om (M.S.A.-A.); sanaullah@unizwa.edu.om (S.U.); 2 School of Health Science, University of Petroleum and Energy Studies, Dehradun 248007, India; 3 Centre for Transdisciplinary Research, Department of Pharmacology, Saveetha Institute of Medical and Technical Science, Saveetha Dental College, Chennai 600077, India; 4 Department of Pharmaceutics, College of Pharmacy, Prince Sattam Bin Abdulaziz University, Al-kharj 11942, Saudi Arabia; m.anwer@psau.edu.sa (M.K.A.); moh.aldawsari@psau.edu.sa (M.F.A.); 5 Department of Food Engineering, Adana Alparslan Turkes Science and Technology University, Adana 01250, Turkey; esrakoca.tr@outlook.com (E.K.); lyaydemir@atu.edu.tr (L.Y.A.)

**Keywords:** sodium alginate, casein, sage essential oil, edible films, food packaging, antioxidant activity

## Abstract

The aim of this study was to examine the effect of Sage (*Salvia sclarea*) essential oil (SEO) on the physiochemical and antioxidant properties of sodium alginate (SA) and casein (CA) based films. Thermal, mechanical, optical, structural, chemical, crystalline, and barrier properties were examined using TGA, texture analyzer, colorimeter, SEM, FTIR, and XRD. Chemical compounds of the SEO were identified via GC–MS, the most important of which were linalyl acetate (43.32%) and linalool (28.51%). The results showed that incorporating SEO caused a significant decrease in tensile strength (1.022–0.140 Mpa), elongation at break (28.2–14.6%), moisture content (25.04–14.7%) and transparency (86.1–56.2%); however, WVP (0.427–0.667 × 10^−12^ g·cm/cm^2^·s·Pa) increased. SEM analysis showed that the incorporation of SEO increased the homogeneousness of films. TGA analysis showed that SEO-loaded films showed better thermal stability than others. FTIR analysis revealed the compatibility between the components of the films. Furthermore, increasing the concentration of SEO increased the antioxidant activity of the films. Thus, the present film shows a potential application in the food packaging industry.

## 1. Introduction

Edible films prepared from natural polymers offer various benefits, including biodegradability, sustainability, and non-toxicity; they also enhance the nutritional quality, shelf life, and safety of the packed food [1]. The selection of polymer for the fabrication of edible films is the most critical phase in attaining desirable packaging and preservation properties. The film-forming properties of sodium alginate, such as transparency and mechanical characteristics, make it a suitable material for preparing edible films [2]. Moreover, casein (CA)-based edible films possess good strength and low oxygen permeability, but due to their hydrophilic nature, they are more sensitive to moisture [3]. Various studies have demonstrated that the cross-linking of natural polymers is a crucial process that enhances the mechanical and physical properties of edible films to make them more competitive compared to traditional packaging material [4].

Sodium alginate is a hydrophilic macromolecule; as a result, this macromolecule provides very little barrier to the passage of water vapors. Adding hydrophobic ingredient such as an essential oil and casein to polysaccharide based films could be an effective way to enhance the films’ morphological, mechanical, thermal, barrier, and antioxidant properties [5]. Essential oils received immense attention as a replacement for synthetic additives since they are generally recognized as safe (GRAS) ingredients by the Food and Drug Administration [6]. *Salvia sclarea*, also called Clary sage, is an aromatic plant that is a member of the *Lamiaceae* family, one of the most important groups of medicinal plants [7]. Sage essential oil (SEO) contains various important chemical constituents, such as linalyl acetate, linalool, terpineol, geranyl acetate, and neryl acetate [8]. Different studies have demonstrated the medicinal and food applications of SEO as an anti-inflammatory [9], antibacterial [7], antioxidant [10], viricidal [11] and natural preservative agent [12].

According to several studies, the incorporation of essential oils into edible films improves their barrier, antimicrobial, and antioxidant properties [5]. Cozmuta et al. [13] prepared and characterized edible films loaded with SEO. The films showed a decreased water vapor permeability (from 16.85 to 2.15 × 10^−10^ g^−1^ m^−1^ Pa^−1^) and moisture content (from 9.90 to 8.35 g water/100 g of dry film). Ehsani et al. [12] examined the antioxidant potential of films loaded with SEO; the findings demonstrated that films exhibited significant antioxidant activities. It’s important to investigate the effect of SEO addition over the physiochemical characteristics of composite films based on SA–CA. Therefore, the current study aims to examine the physiochemical properties of SA–CA-based edible films loaded with SEO.

## 2. Results and Discussion 

### 2.1. GC–MS Analysis of Sage Essential Oil

**Figure 1:**
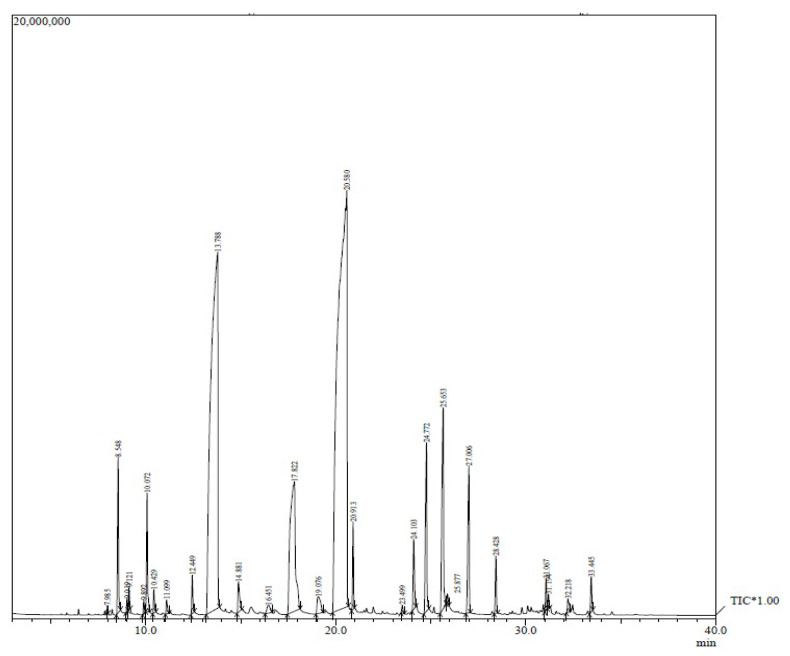
GC–MS analysis of the sage essential oil.

The presence of different components in SEO was confirmed by GC–MS (Figure 1). The primary components found in SEO were linalyl acetate, linalool, terpineol, geranyl acetate, and neryl acetate (Table 1).

Linalyl acetate and linalool are the primary components of many essential oils that possess therapeutic potential, such as anti-inflammatory qualities [14]. The results of the current study are in line with Wu et al. [15], where linalyl acetate (46.63%) and linalool (22.68%) were identified as major components in SEO, followed by geranyl acetate and terpineol. Moreover, Cai et al. [8] also reported similar results, in which chemical analysis of SEO revealed the presence of linalyl acetate (49.83%), linalool (28.76%), terpineol (5.05%), geranyl acetate (2.79%), along with neryl acetate (1.61%).

The chemical composition of SEO show variation depending on various factors, such as the origin of the plant, growing season, weather conditions, time of harvest, and humidity. The time period between the harvest of the raw material and the oil extraction is another crucial factor that can affect the chemical composition of the oil.

### 2.2. Visual Characterization

**Figure 2:**
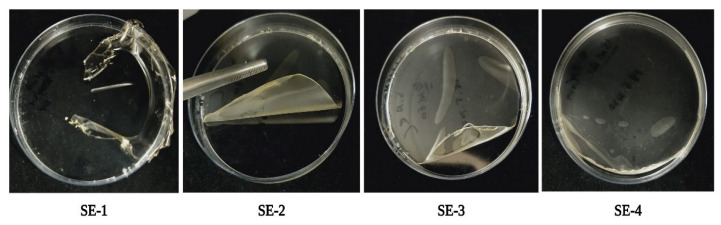
Visual characterization of different samples of SA–CA-based edible films. Control or SE-1: SA + CA; SE-2: SA + CA + SEO 5 μL; SE-3: SA + CA + SEO 10 μL; and SE-4: SA + CA + SEO 15 μL.

It is important to characterize edible films based on visual properties to analyze their physical characteristics. These characteristics include transparency, mechanical properties, fragility, flexibility, etc. [16]. Samples of the prepared edible films based on SA and CA were analyzed for their visual parameters (Figure 2). It was observed that the SE-1/control with no SEO addition was transparent and comparatively more fragile when compared to the films loaded with SEO. The films with SEO addition showed good characteristics in terms of flexibility, brittleness, and stiffness. The SE-4 sample with 15 μL SEO showed less transparency and more flexibility compared to SE-2 and SE-3. This could be due to the incorporation of SEO, resulting in changes in the film matrix.

### 2.3. Thickness

The thickness results of the prepared SA–CA-based edible film samples showed non-significant results. There was no significant difference between the thickness of the control film samples and the samples loaded with SEO. Hashemi et al. [17] also reported similar results in which the addition of oregano essential oil did not affect the thickness of the Basel seed gum edible films.

### 2.4. Mechanical Properties

The variation in mechanical characteristics of biopolymer-based films is based on polymer type, manufacturing methods, thickness of the film, and exposure to factors that causes structural changes in polymers, such as heat, moisture and light. The extent of the variation depends on the composition of the edible film. It is important to optimize the proportion of the ingredients of the composite films to develop an appropriate stable packaging material.

The results obtained for the mechanical parameters of the SA–CA-based edible films are shown in Table 2. The results showed a decrease in both tensile strength (TS) and elongation at break (EAB) of film samples with an increase in the concentration of SEO. The TS and EAB values of the SE1 sample were found to be higher when compared to samples loaded with SEO. The TS and EAB values for SE-4 (15 μL of SEO) were found to be lower than SE-3 and SE-2 with 10 μL and 5 μL of SEO, respectively. The decrease in the tensile strength and EAB could be attributed to the addition of oil, resulting in weaker intermolecular interactions between oil and polymers compared to the interaction between only polar molecules of control sample. This behavior could also be ascribed to the decrease in the crystalline structure of the film with the addition of SEO, resulting in reduced TS and EAB. 

In our previous work, a decrease in tensile strength was observed when ginger essential oil was added to chitosan and porphyrin-based composite films [16]. Jamróz et al. [18] also reported similar results in which the addition of tea tree essential oil decreased the TS and EAB values of the starch–furcellaran–gelatin films.

### 2.5. Moisture Content and Water Solubility

Biopolymer-based films should be water resistant to prevent food spoilage. Polysaccharides-based edible films have poor water resistance, and thus, it is important to improve their water resistance by incorporating nonpolar substances. In the current study, the moisture content of oil-loaded SA–CA-based edible films were determined, and the findings are expressed in Table 2. The control (SE-1) film sample without oil exhibited maximum moisture content (25.04%) compared to film samples incorporated with SEO. With the addition of SEO in the films, the moisture content decreased (Table 2). The SE-4 sample with 15 μL of SEO addition showed minimum moisture content, followed by SE-3 and SE-2 with 10 μL and 5 μL SEO addition, respectively. This could be due to the hydrophobic nature of the oil present in the films. As per the previous work, sodium alginate and casein-based films showed similar behavior when incorporated with orange essential oil [5]. Moreover, the water solubility results showed that all the film samples were soluble in the water. This behavior may be due to the hydrophilic nature of the sodium alginate, as a small amount of oil added to the films did not significantly affect their hydrophilicity.

### 2.6. Water Vapor Permeability

The microstructural characteristics of film directly affect the WVP of the films. The results obtained for the water vapor permeability of the SA–CA-based edible films are presented in Table 2. The findings showed an increase in the water vapor permeability of the film samples with an increase in the concentration of incorporated SEO. The control film sample showed less water permeability compared to the SEO-loaded film samples. The maximum water vapor permeability was observed in the SE-4 sample with 15 μL of SEO addition, followed by SE-3 and SE-2 with 10 μL and 5 μL SEO addition, respectively. The increase in the WVP could be due to the irregular distribution of SEO in the film, resulting in a heterogeneous structure. The cracks and pores in the SE-4 sample may be the reason for the increased water vapor permeability (Figure 5). The ratio of the hydrophilic and hydrophobic film-forming components also affects the water permeability. Ahmad et al. [19] found the same results in which the WVP of the gelatin films increased with an increase in the amount of bergamot essential oil.

### 2.7. Transparency

Transparency and color attributes are the most important aspects of polymer-based films that affect the overall appearance and consumer acceptability. The findings for the transparency of the film samples examined are shown in Table 3. The transparency of the SA–CA-based edible film decreased with increase in the concentration of SEO. The control sample showed maximum transparency compared with SEO-loaded edible film samples. The lowest transparency was observed in SE-4 films with 15 μL of SEO incorporated, followed by SE-3 with 10 μL of SEO and SE-2 with 5 μL SEO incorporated. The decrease in transparency may be due to the addition of SEO, which contains different color components. The structural changes in the film matrix caused by the incorporation of oil also affect the transparency of the film samples. Tongnuanchan et al. [20] also observed a reduction in the transparency of gelatin-based edible films with the incorporation of palm oil. Moreover, the results of the current study are also in agreement with the findings of Scartazzini et al. [21], in which a decrease in transparency was observed when films were loaded with mint essential oil.

### 2.8. Color Parameters

The color of the biopolymer-based films is an important characteristic in terms of their appearance and consumer acceptability. In the current study, different SA–CA-based edible film samples were examined for color parameters, and the results are presented in Table 3. All the film samples exhibited similar lightness (L*) values. The observations of the current study suggest that adding SEO significantly impacted the color and appearance of the films. The control films showed a more transparent appearance compared to SEO-loaded films. The SEO slightly altered the color of films from transparent to pale yellow (b*), as this can be observed in Table 3. The reduction in the transparency of SEO-loaded film samples could be ascribed to the different color components present in the oil. The results for the color parameters are consistent with visual observations of the films. Moreover, a slight decrease in the lightness and variations in the color parameters of the whey protein-based films was also observed with the incorporation of rapeseed oil [22].

### 2.9. TGA

**Figure 3:**
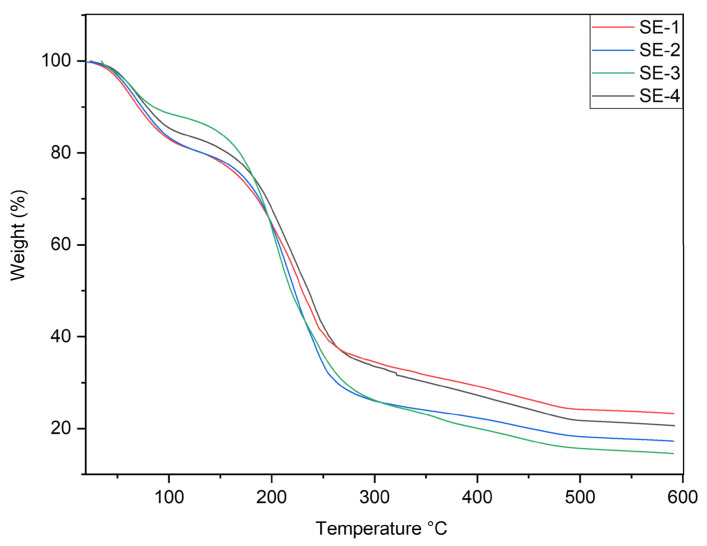
TGA analysis of different samples of SA–CA-based edible films. Control of SE-1: SA + CA; SE-2: SA + CA + SEO 5 μL; SE-3: SA + CA + SEO 10 μL; and SE-4; SA + CA + SEO 15 μL.

The thermal stability of film samples was studied via thermogravimetric analysis. In the range of temperature of 25–600 °C, all samples showed patterns of thermal degradation with several stages, as shown in Figure 3. The first phase of thermal degradation occurred between 35–90 °C with 12% weight loss. This could be due to the evaporation of bounded water and SEO present in the film samples [5]. The next phase of thermal degradation occurred between 120–400 °C. In this phase, the maximum weight loss approximately 80% of the film samples appeared. The reduction of weight in this phase could be due to the thermal degradation of the film-forming components, including SA, CA, and glycerol. 

The SE-3 and SE-4 films showed good thermal stability compared to SE-2 and control. This could be attributed to the addition of SEO in the films resulting in enhanced thermal stability, as shown in Figure 3. In our earlier work, sodium alginate and casein-based film samples showed good thermal properties when loaded with orange essential oil [5].

### 2.10. XRD

**Figure 4:**
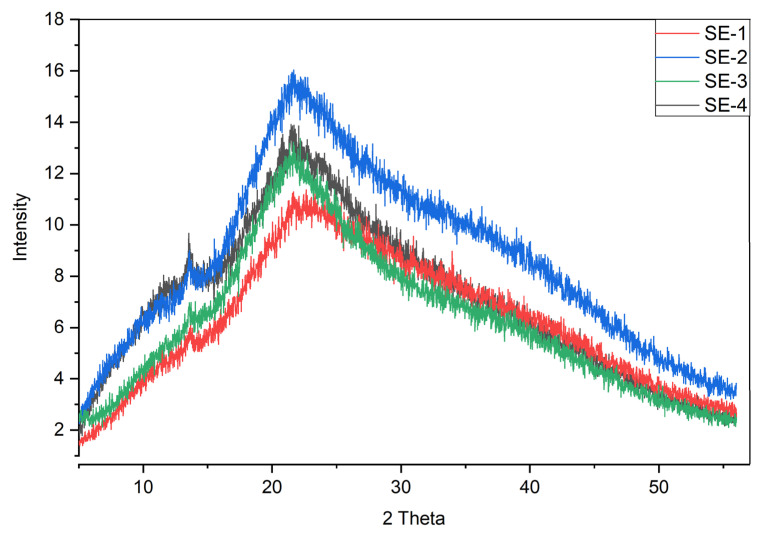
XRD characterization of different samples of SA–CA-based edible films. Control or SE-1: SA + CA; SE-2: SA + CA + SEO 5 μL; SE-3: SA + CA + SEO 10 μL; and SE-4; SA + CA + SEO 15 μL.

X-ray diffraction analysis was carried out to examine the structures of the different samples of the prepared films based on SA–CA. The findings from XRD revealed that all the film samples showed characteristic diffraction peaks at different angles, presenting a semi-crystalline structure. After calculating the crystallinity by Diffract Eva software package, the crystallinity of the SE-1, SE-2, SE-3, and SE-4 film samples was found to be 13.3%, 12.9%, 11.3%, and 9.3%, respectively. A slight decrease in the crystallinity of the film samples was observed with the addition of SEO. This behavior could be due to the hydrophilic nature of the polymers resulting in increased moisture content in the films avoiding forming a crystalline structure. In our previous work, similar findings were obtained for sodium alginate and casein films incorporated with orange essential oil [5]. Moreover, similar structural behavior of the sodium alginate and casein films was observed by Bora and Mishra [23]. Furthermore, the variation in the peak intensity could be ascribed to the difference in the concentrations of the SEO added to film samples. The XRD characterization of different samples of SA–CA-based edible films is shown in Figure 4.

### 2.11. SEM Analysis

**Figure 5:**
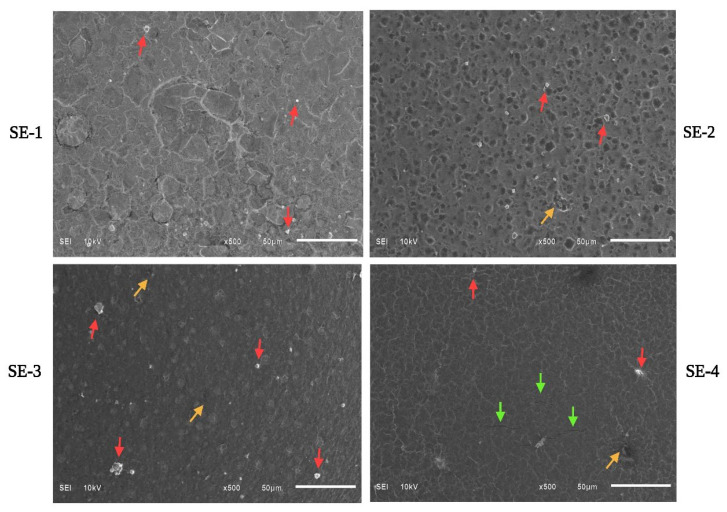
SEM examination of different samples of SA–CA-based edible films. Control or SE-1: SA + CA; SE-2: SA + CA+ SEO 5 μL; SE-3: SA + CA + SEO 10 μL; and SE-4; SA + CA+ SEO 15 μL. Red arrows for particles on the surface. Yellow arrows for pores, and green arrows for cracks on the surface.

The microscopic structural changes in the fabricated composite films were assessed by SEM [24]. The obtained results are presented in Figure 5. The SE-1 or control film sample showed a rough surface with fewer particles. In our previous study, SA–CA-based control films showed similar structural morphology having rough, bulged surfaces with many particles [5]. The surface of SE-2 and SE-3 films was coarser with several voids and particles compared to control and SE-4. This could be due to the non-homogenous mixing of film-forming constituents, which led to irregularities in the developed film structures.

SEM of the film sample of SE-4 with SEO (15 μL) revealed a smooth surface but with a few particles, pores, and cracks, as shown in Figure 5. Overall, the film samples loaded with SEO exhibited heterogeneous structures with more pores and cracks; this indicates the weak interaction between oil and polymers in the film matrix. In our previous work, the similar observations were noticed for sodium alginate and casein-based edible films loaded with orange essential oil [5]. Similar observations were also reported by Pereda et al. [25] when chitosan-based films were incorporated with olive oil. The film microstructure behavior can also be affected by the drying process and conditions.

### 2.12. Fourier Transform Infrared Spectroscopy Spectrum (FTIR) Analysis

**Figure 6:**
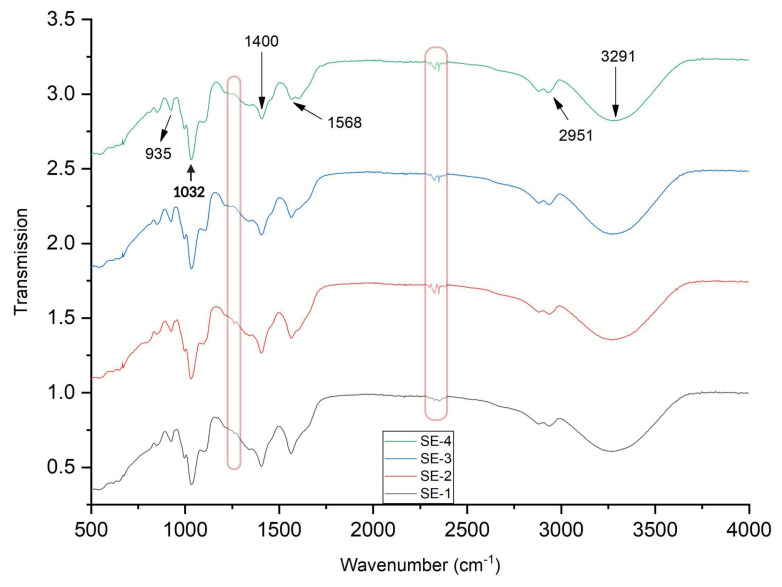
FTIR analysis of different samples of SA–CA-based edible films. Control of SE-1: SA + CA; SE-2: SA + CA+ SEO 5 μL; SE-3: SA + CA + SEO 10 μL; and SE-4; SA + CA + SEO 15 μL.

To investigate the intermolecular interactions between sodium alginate, pectin, glycerol, and SEO, Fourier transform infrared spectroscopy spectrum was performed. Figure 6 presents the FTIR spectrum of the SEO-loaded film samples. The characteristic peaks at 2951 cm^−1^ present the C–H stretching of the alkane groups, while the peak band at 935 cm^−1^ represents the C–O stretching vibration. The characteristic peak at 1400 cm^−1^ indicates the O–H bending of carboxylic. Previous studies indicated that peaks at 2952, 1400, and 935 cm^−1^ could be ascribed to the presence of sodium alginate [5,26].

The spectra in Figure 6 show a peak at 3291 and 1032 cm^−1^ caused by the stretching vibration of N–H and the strong stretching of the S=O group, respectively. It was reported in the literature that characteristic peaks at 3291 and 1031–1001 cm^−1^ could be due to casein present in the sample [5,27]. The characteristic peat at 1568 cm^−1^ represents the medium C=C stretching of the alkene. A smaller shift in the wavenumber of the film samples could be ascribed to the different concentrations of the SEO. The broad peak in the range of 3600–3200 cm^−1^ corresponded to the –OH stretching vibrations and hydrogen bonding among hydroxyl groups of glycerol, water, and emulsified oil droplets [28]. In our previous study, the CA–SA-based edible films showed a similar spectrum in FTIR analysis [5]. The slight changes with the formation of characteristic peaks in oil-loaded samples were observed at 1250 and 2350 cm^−1^. A peak at 1250 cm^−1^ could be due to the presence of linalyl-acetate, possibly induced by the ester C−O stretch [29]. Furthermore, FTIR analysis proved that the addition of oil to films did not alter the chemical composition as much. This phenomenon showed that no chemical reaction occurred and that the chemical structure of the resulting films was completely stable. Instead of the formation of new peaks due to the chemical reaction, a change in the intensities of FTIR was observed, which could be due to the change in the hydrophobicity of the films [30]. Furthermore, similar FTIR patterns have also been reported in previous reports, where the addition of oil and other additive had not caused a significant difference in the spectra [31,32,33]. Thus, FTIR spectra results showed incorporating the films with oil did not show major intermolecular interaction or structural alteration in the film matrix.

### 2.13. Antioxidant Activity

**Figure 7:**
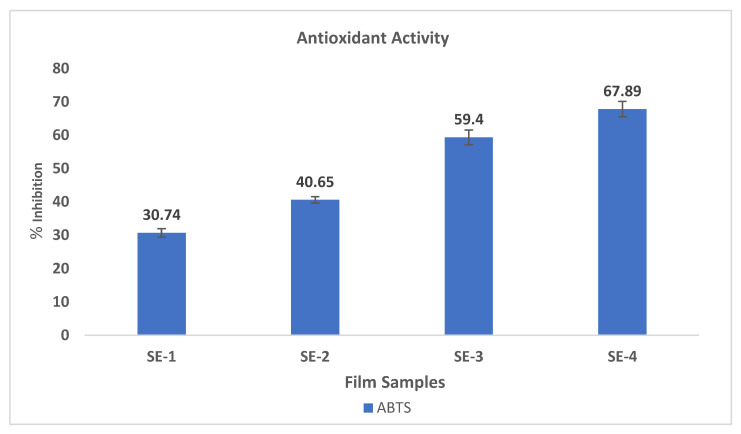
Antioxidant activity of SA–CA-based edible film samples. Control of SE-1: SA + CA; SE-2: SA + CA + SEO 5 μL; SE-3: SA + CA + SEO 10 μL; and SE-4; SA + CA + SEO 15 μL.

The addition of natural antioxidants, such as essential oil, can inhibit the oxidation of various food components, such as lipids and proteins. In the current study, the SEO was added to the edible films to enhance their antioxidant activity, resulting in the improvement of the stability, shelf life, and overall quality of the food. The antioxidant activity of the SA–CA-based edible film samples was examined, and the results are depicted in Figure 7. The control sample (SE-1), without any addition of SEO, showed low ABTS radical scavenging activity compared to film samples incorporated with essential oil. The SE-4 sample loaded with 15 μL of SEO showed the highest ABTS radical scavenging activity compared to samples with 10 μL and 5 μL of SEO. This increase in antioxidant activity with an increase in the concentration of oil could be due to the presence of different phenolic compounds in SEO. Bozin et al. [34] studied the antioxidant potential of SEO, and the results demonstrated that this oil possesses excellent radical scavenging activity. Furthermore, various studies have demonstrated the increase in the antioxidant activity of different biopolymer-based edible films when incorporated with essential oils.

In our previous work, the chitosan–porphyrin composite films showed increased antioxidant activity when incorporated with ginger essential oil [20]. Our findings are in agreement with the findings of Ruiz-Navajas et al. [35], where an increase in the antioxidant activity of chitosan-based edible films was observed with the incorporation of Thymus piperella essential oil.

## 3. Conclusions

In the current study, SEO was used to fabricate edible films based on SA and CA composite. The incorporation of SEO influenced various properties of the film matrix, including morphological structure. In conclusion, addition of SEO reduced the TS, EAB, MC and transparency while WVP, thermal stability and antioxidant activity were increased with the incorporation of SEO. It was found that films possessed antioxidant effects. It is anticipated that by utilizing this new oil-loaded composite film, the quality and safety of food materials can be maintained. The SA–CA-based edible films loaded with SEO have the potential to be used in the food packaging industry.

## 4. Materials and Methods

### 4.1. Chemical Procurement

Sage (*Salvia sclarea*) essential oil was procured from Nature Natural, Ghaziabad, India. Sodium alginate (pure) and casein were obtained from Sisco Research Laboratories Pvt Ltd., Mumbai, India. The glycerol used in this study for film formation was supplied by BDH Laboratory, London, UK. Additionally, other required chemicals, such as 2,2′-diphenyl-1-picrylhydrazyl, (DPPH) ABTS (2,2′-azinobis-(3-ethylbenzothiazoline-6-sulfonic acid), butylated hydroxyl anisole, and Trolox (6-Hydroxy-2,5,7,8-tetramethylchromane-2-carboxylic acid), were provided by the Sigma–Aldrich (St. Louis, MO, USA).

### 4.2. Sodium Alginate and Casein-Based Film Formation

The edible films containing sodium alginate (SA) and casein (CA) were fabricated by following the casting method. Four samples of edible films coded as SE-1-SE-4 were prepared. Initially, a 1.5% solution of sodium alginate and a 1% solution of casein were prepared separately by dissolving the polymers in distilled water. The resultant solutions were mixed thoroughly overnight at 25 °C by a magnetic stirrer. After mixing SA and CA, the solution was evenly divided into four beakers labeled as SE-1, SE-2, SE-3, and SE-4. The SE-1 sample was used as a control in which the composition of the film-forming components was sodium alginate (1.5%), casein (1%), glycerol (0.5%), and sorbitol (0.1%) without the addition of SEO. Different concentrations (5 μL, 10 μL, and 15 μL) of SEO were added to SE-2, SE-3, and SE-4 sample, respectively. The composition of the film-forming solution is shown in Table 4. After preparations and mixing of the components, the solutions (20 mL) for each sample were poured onto the labeled petri plates (90 mm × 15.8 mm) for drying at 25 °C for 48 h. After performing visual inspections of the fabricated films, the samples were peeled from the Petri plate surface, kept in desiccators for 12 h at 24 °C, and subjected to further analysis.

### 4.3. GC–MS of the SEO

GC–MS analysis was performed to determine the chemical composition of SEO. SEO was subjected to GC–MS analyses using a Shimadzu, Tokyo, Japan, GCMS-QP-2010 Plus Gas Chromatograph Mass Spectrometer. The column was kept at 50 °C for 2 min after injection before being programmed at 5 °C/min to 210 °C and then at 10 °C/min to 280 °C. With a split ratio of 1:115, the injection volume was 1.0 l of pure essential oil. The carrier gas was helium, and the flow rate was maintained at 1.0 (mL/min). The temperature of the injector was 260 °C, and the temperature of the ion source was 220 °C. The compounds were recognized by contrasting the retention times and retention indices of the chromatographic peaks with those of authentic reference standards that were subjected to the exact same conditions during the chromatographic analysis. The National Institute of Standards and Technology (NIST) MS spectral database (version 2005) was used to search the mass spectrum database and to compare the MS fragmentation pattern with those of pure compounds.

### 4.4. Thickness of the Films

The SA–CA-based edible films were evaluated for thickness measurements using a micrometer (Mitutoyo digital micrometer 2046F, Mitutoyo, Kawasaki, Japan). The average value for the thickness was calculated by taking five random measurements of different areas of the analyzed film samples.

### 4.5. Mechanical Properties of the Films

The desirable mechanical characteristics of edible films are essential because they act as an indicator of the durability and cohesiveness of the films [14]. In the current study, an ASTM D882 (American Society for Testing and Materials. ASTM., 2010) standardized method was employed to measure the mechanical strength of the SA–CA-based edible films. A universal tester (TA. XT plus, Stable Micro Systems, London, UK) was utilized for conducting the test. The fabricated edible films were allowed to be conditioned for a minimum of 40 h in a test cabinet (Nüve TK 120, Ankara, Turkey). To examine the mechanical characteristics of the films, the following measurements were taken:

(1) $$Tensile\;Strenght\;(TS)=(\frac{F}{A})$$

where *F* denotes the force, and *A* shows the film’s cross-sectional area.

(2) $$Elongation\;at\;break\;(EAB)\;\left(\%\right)=\frac{Lf-Li}{Li}\times 100$$

where *Li* displays the film’s initial length, and *Lf* indicates the final length at a break.

### 4.6. Moisture Content

The percentage of moisture in samples of the prepared SA–CA-based edible films was assessed by the gravimetric method. In this method, the films were dried at 105 °C, and the weight of the films before and after drying was measured as *W*1 and *W*2, respectively. The moisture content was determined by the following formula:

(3) $$Moisture\;content=\frac{W1-W2}{W1}\times 100$$

### 4.7. Water Solubility

For water solubility, the methodology of Kim and Song [36] was adopted. The films were dried at 105 °C and weighed as *W*1. After diluting with 20 mL of distilled water, the films were kept in the shaking incubator for 24 h. After this, the samples were again dried in the oven at 105 °C and weighed as *W*2. Following the equation, the water solubility of SA–CA-based edible film samples was determined.

(4) $$Water\;solubility=\frac{W1-W2}{W1}\times 100$$

### 4.8. Water Vapor Permeability (WVP) of the Films

The WVP of the SA–CA-based film samples were evaluated gravimetrically by following the methodology of Erdem et al. [37]. The relative humidity in the desiccator that contained the films was brought down to 50%. In the process, we used glass cups that had a diameter and depth of 5 cm and 3 cm, respectively. The relative humidity (RH) of the measuring systems was regulated by using water and silica gel having RH of 100% and 0%, respectively. To evaluate weight gain during the day, cups containing silica gel were covered with films that were firmly sealed and periodically weighed every hour. WVP was calculated using the formula and presented in g mm/(m^2^) (d) (kPa):

(5) $$Water\;vapor\;permeability=\frac{\Delta m}{\Delta t\times \Delta P\times A}\times d$$

where ∆*m*/∆*t* is the moisture gain per unit of time in g/d, *A* is the film area in m^2^, ∆*P* is the difference in the water vapor pressure between both sides of the film in kPa, and *d* indicates the film thickness.

### 4.9. Transparency

The transparency of the film samples was evaluated via a spectrophotometer, according to the method of Zhao et al. [38]. The spectrophotometer was set to 550 nm, and the absorbance of the rectangular film samples was measured after being placed in Spectro cuvettes. 

### 4.10. Color Analysis

Color parameters are very important factors in determining consumer acceptability and the overall appearance of the edible films. The prepared film samples were examined for color analysis using a colorimeter (Konica Minolta, Tokyo, Japan). The a* (red–green) and b* (yellow–blue) parameters were assessed with a reference plate (L* = 100). The color analysis was performed at various positions on the films, and the results were calculated using the following Equation:

(6) $$\Delta E*={{\left[(\Delta L*\right)}^{2}+{\left(\Delta a*\right)}^{2}+{\left(\Delta b*\right)}^{2}]}^{1/2}$$

The overall color difference is denoted by the symbol *E*.

### 4.11. Thermogravimetric Analysis

The thermal stability of the SA–CA-based edible films was determined via a thermal gravimetric analyzer (TA instruments, New Castle, DL, USA). The temperature range was 25–600 °C, and the film samples were scanned at a rate of 10 °C per min.

### 4.12. XRD

The X-ray diffraction methodology was used to examine the extent of crystallinity and amorphousness of the SA–CA-based edible films. The XR-Diffractometer Bruker D8 Discover instrument was used, and it was configured to operate with 2θ ranging from 5 to 50° at 10 kV.

### 4.13. Scanning Electron Microscopy

The prepared samples SE-1 to SE-4 of the SA–CA-based edible films were evaluated by SEM at 10kv using a JSM6510LA from Analytical SEM, Jeol, Japan, to study the surface morphology as well as the cross-sectional structural properties of the films. The samples were manually fractured and then the tape was used to attach them to the aluminum ends. Then film samples were coated with a layer of gold before taking images.

### 4.14. Fourier Transform Infrared Spectroscopy Analysis

FTIR was used to identify the functional groups present in the edible film, as well as to study the interactions between different components of the film, such as the polymer matrix and SEO. The FTIR analysis was performed by InfraRed Bruker Tensor 37, Ettlingen, Germany. The test was performed at 25 °C, with an average of 32 scans. The range of wavenumber was from 500–4000 cm^−1^.

### 4.15. Antioxidant Activity

The methodology of Re et al. [39] was adopted with a few modifications for the assessment of ABTS radical scavenging activity of the SA–CA film samples (3.125 mg). The change in the absorbance was measured at 734 nm. The results for the antioxidant activity of the film samples were measured and presented as % inhibition.

### 4.16. Statistical Analysis

All data in the current study is reported as the mean, standard deviation (SD) of three distinct assessments. A one-way analysis of variance was conducted using statistical analysis software, followed by Duncan’s test with a 5% significant level. 

## Figures and Tables

**Table 1:** Primary components detected in sage essential oil.

No.	Name	R. Time	Composition %
1	Linalool	13.78	28.51
2	Terpineol	17.822	8.09
3	Linalyl acetate	20.580	43.32
4	Geranyl acetate	25.653	4.04
5	Neryl acetate	24.772	2.85
6	Caryophyllene	27.006	2.44
7	Myrcene	8.548	1.85

**Table 2:** TS, EAB, thickness, WVP, and moisture content of the SA–CA-based edible films.

Sample Code	TS (Mpa)	EAB (%)	Thickness (mm)	WVP (×10^−12^ g·cm/cm^2^·s·Pa)	Moisture Content (%)
SE-1	1.022 ± 0.051 ^a^	28.2 ± 0.643 ^a^	0.053 ± 0.007 ^a^	0.427 ± 0.009 ^a^	25.04 ± 0.47 ^a^
SE-2	0.823 ± 0.043 ^b^	21.9 ± 1.30 ^b^	0.050 ± 0.014 ^a^	0.485 ± 0.007 ^b^	22.53 ± 1.03 ^b^
SE-3	0.576 ± 0.029 ^c^	14.8 ± 0.412 ^c^	0.045 ± 0.015 ^a^	0.537 ± 0.034 ^c^	18.38 ± 1.30 ^c^
SE-4	0.140 ± 0.014 ^d^	14.6 ± 0.06 ^c^	0.053 ± 0.007 ^a^	0.667 ± 0.022 ^d^	14.70 ± 0.37 ^d^

Means carrying the same letters are significantly identical.

**Table 3:** Color attributes and transparency of the SA–CA-based edible film samples.

Sample Code	*L*	a*	b*	ΔE*	Transparency %
SE-1	97.50 ± 0.11 ^a^	−0.13 ± 0.02 ^a^	2.63 ± 0.11 ^a^	2.28 ± 0.15 ^a^	86.100 ± 0.561 ^a^
SE-2	97.12 ± 0.14 ^a^	−0.26 ± 0.03 ^b^	4.35 ± 0.37 ^b^	3.63 ± 0.36 ^b^	82.319 ± 0.402 ^b^
SE-3	97.65 ± 0.14 ^a^	−0.28 ± 0.01 ^b^	4.62 ± 0.20 ^b^	4.07 ± 0.14 ^c^	74.560 ± 0.607 ^c^
SE-4	97.59 ± 0.56 ^a^	−0.26 ± 0.04 ^b^	5.55 ± 0.14 ^c^	4.93 ± 0.24 ^d^	56.299 ± 0.092 ^d^

*L*: lightness, a*: green-red color, b*: blue-yellow color, ΔE*: overall color variation. Means carrying the same letters are significantly identical (*p* ≥ 0.05).

**Table 4:** Chemical composition of the film-forming solutions for different samples.

Film Sample	The Composition of the Film-Forming Solution
SE-1/Control	SA (1.5%) + CA (1%) + Gly (0.5%) + Sorb (0.1%)
SE-2	SA (1.5%) + CA (1%) + Gly (0.5%) + Sorb (0.1%) + SEO (0.025%)
SE-3	SA (1.5%) + CA (1%) + Gly (0.5%) + Sorb (0.1%) + SEO (0.050%)
SE-4	SA (1.5%) + CA (1%) + Gly (0.5%) + Sorb (0.1%) + SEO (0.075%)

SA: Sodium alginate; Gly: glycerol; Sorb: sorbitol; CA: casein; SEO: Sage essentials oil.

## Data Availability

Not applicable.
